# Time‐Dependent Diagnostic Yield and Therapeutic Impact of Implantable Loop Recorders, Brugada Syndrome as the Exception

**DOI:** 10.1111/jce.70469

**Published:** 2026-09-06

**Authors:** Luigi Cocchiara, Benedetta Brescia, Procolo Marchese, Stefano Nardi, Luigi Argenziano, Gianmarco Arabia, Marco Micillo, Francesca Gennaro, Giovanni Mazzotta, Lucio Addeo, Alessandro Landolfi, Emiliano Calvi, Manuel Cerini, Alessio Nicolai, Teresa Strisciuglio, Pasquale Vergara, Giovanni Esposito, Antonio Rapacciuolo

**Affiliations:** ^1^ Department of Advanced Biomedical Sciences, Division of Cardiology Federico II University of Naples Naples Italy; ^2^ Cardiology, Heart Department Pineta Grande Hospital Naples Italy; ^3^ EP Lab Cardiology Mazzoni Hospital, AST Ascoli Piceno Italy; ^4^ Division of Cardiology Spedali Civili Hospital Brescia Italy; ^5^ U.O. Sincope Unit IRCCS Neuromed Pozzilli Italy; ^6^ Betania Evangelical Hospital Naples Italy

**Keywords:** atrial fibrillation, Brugada syndrome, cryptogenic stroke, implantable loop recorder, palpitations, syncope, time to diagnosis, ventricular arrhythmia

## Abstract

**Introduction:**

The optimal duration of implantable loop recorder (ILR) monitoring remains uncertain and may differ according to the indication for implantation. We evaluated whether the time‐dependent diagnostic yield and clinical relevance of ILR‐detected arrhythmias differ among patients implanted for syncope, palpitations, cryptogenic stroke, or Brugada syndrome.

**Objective:**

This study sought to define the time‐dependent diagnostic yield of ILRs across clinical indications and to assess whether early and late diagnoses differed in their impact on clinical management.

**Methods and Results:**

We retrospectively analyzed 1734 consecutive patients undergoing ILR implantation at 5 Italian centers for unexplained syncope, palpitations, cryptogenic stroke, or Brugada syndrome. Diagnosis of interest was defined according to the implant indication: pauses for syncope, supraventricular tachycardia or atrial fibrillation for palpitations, atrial fibrillation for cryptogenic stroke, and ventricular tachycardia or pauses for Brugada syndrome. Median follow‐up was 34 months. Maximally selected rank statistics identified 26 months as the global threshold separating early from late diagnoses, while indication‐specific optimal diagnostic windows were 14 months for syncope, 13 months for palpitations, 16 months for cryptogenic stroke, and 55 months for Brugada syndrome. In syncope and palpitations, most actionable diagnoses occurred early, and diagnostic efficiency declined markedly after 26 months, with late findings often less clearly related to the index presentation. In cryptogenic stroke, atrial fibrillation detection was most frequent early but could still lead to changes in antithrombotic management later, supporting an individualized “gray‐zone” approach to prolonged monitoring. By contrast, Brugada syndrome showed sustained diagnostic yield over extended follow‐up, with late ventricular arrhythmias or pauses retaining major prognostic and therapeutic implications, including implantable cardioverter‐defibrillator implantation and device‐type selection.

**Conclusions:**

The value of ILR monitoring is strongly time‐ and indication‐dependent. Prolonged routine monitoring or reimplantation appears to have limited incremental value in syncope and palpitations when early follow‐up is unrevealing, whereas extended surveillance may remain justified in Brugada syndrome and should be individualized after cryptogenic stroke. These observations support indication‐driven ILR follow‐up strategies rather than a uniform approach based on device longevity alone.

## Graphical abstract

Diagnostic yield and therapeutic impact varied substantially according to the indication for implantation. Actionable diagnoses were concentrated in the early follow‐up period for syncope and palpitations, whereas late atrial fibrillation detection after cryptogenic stroke could still modify antithrombotic management. In contrast, patients with Brugada syndrome showed sustained detection of ventricular tachycardia and clinically significant pauses over prolonged follow‐up, supporting extended surveillance. These findings favor indication‐driven monitoring and reimplantation strategies rather than a uniform approach based on device longevity alone.

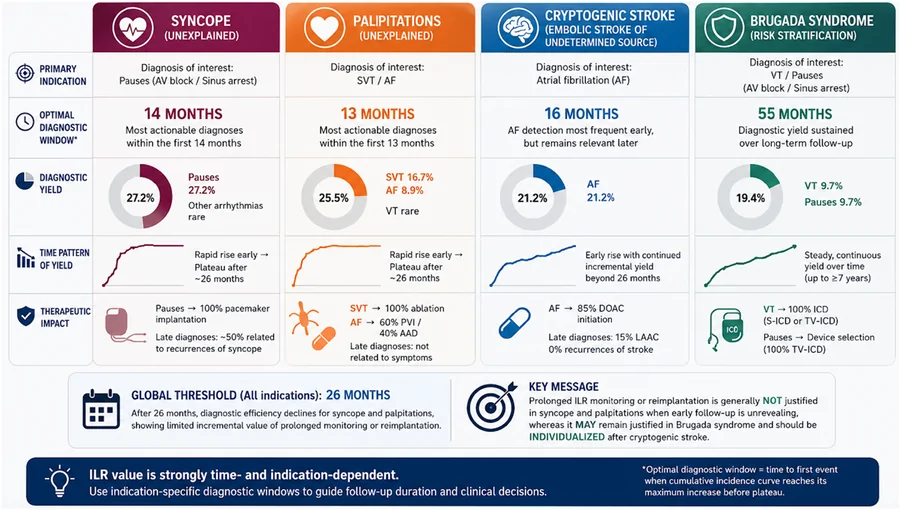

**Abbreviations**
AF: atrial fibrillation
ILR: implantable loop recorder
NNF: number needed to follow (number of patients who need to be monitored for one event)
RM: remote monitoring
SVT: supraventricular tachycardias
VT: ventricular tachycardias

## 1 Introduction

Implantable loop recorders (ILRs) are devices that monitor heart rhythm. Originally developed as a diagnostic tool for unexplained recurrent syncope [1, 2, 3, 4, 5, 6], ILR indications have expanded to include a range of clinical scenarios, including Brugada syndrome, as highlighted by the BruLoop study [7], and atrial fibrillation (AF) detection in patients with cryptogenic stroke or palpitations [8, 9, 10]. Early ILR implantation has been proven to enhance patients' management and overall quality of life [11, 12]. However, up to 70% of cases remain undiagnosed after 3 years of monitoring [8, 13], raising the question of whether monitoring should be extended beyond battery depletion [14]. Only a few studies, most involving limited patient populations, have explored this issue, with follow‐up extending to a maximum of 4 years [15].

Current‐generation ILRs may offer battery longevity of up to 6–7 years, whereas newer models feature a smaller form factor that may come at the expense of shorter battery life.

The clinical value of prolonged monitoring remains insufficiently characterized. Most prior studies have focused primarily on diagnostic yield, without systematically evaluating whether late arrhythmic detections translate into meaningful therapeutic decisions or influence device reimplantation strategies. Understanding the time‐dependent relationship between arrhythmia detection and downstream management is essential to inform rational follow‐up duration, optimize resource utilization, and avoid unnecessary prolonged surveillance. Accordingly, the aim of this study was to define the temporal pattern of ILR diagnostic yield across different clinical indications and to assess the extent to which detected arrhythmias were associated with actionable clinical interventions, thereby informing indication‐driven monitoring strategies.

## 2 Materials and Methods

### 2.1 Study Design

This was a multicenter, retrospective, observational study that included all consecutive patients referred for ILR implantation at five Italian centers. The study was designed and conducted in accordance with the Declaration of Helsinki and followed the Strengthening the Reporting of Observational Studies in Epidemiology reporting guideline. The study protocol was approved by the institutional ethics committees of all participating centers, and all patients provided informed consent.

### 2.2 Patient Population, Data Collection, and Definition Adopted

Patients included in this study had an indication for ILR implantation for syncope, palpitations, cryptogenic stroke, or Brugada syndrome. The pre‐implant diagnostic work‐up varied by clinical indication.

Patients with syncope underwent 12‐lead ECG, transthoracic echocardiogram, 24‐h Holter monitoring, head‐up tilt test, and, at physicians' discretion, cardiac magnetic resonance imaging and electrophysiology study.

Patients with palpitations underwent 12‐lead ECG, transthoracic echocardiogram, 24 h Holter monitoring, and electrophysiology study if deemed necessary by the physicians.

Cryptogenic stroke was defined as an ischemic cerebral event without identifiable cause. Before ILR implantation, patients underwent transthoracic echocardiogram and, according to the centers' protocol, a 24 h Holter monitoring.

Patients with Brugada syndrome received ILR implantation according to the latest ESC guidelines (unexplained syncope) [16] or, according to the centers' protocol, following negative PES in patients with spontaneous or induced type I pattern.

Patients without remote monitoring (RM) capability were excluded from analysis.

“Time to diagnosis” was defined as the interval between ILR implantation and the first appropriately recorded arrhythmic event. “Diagnosis of interest” were defined as the events most likely associated with the implant indication: pauses for patients with syncope, AF for stroke, supraventricular tachycardias (SVT) or AF for palpitations, and ventricular tachycardias (VT) or pauses for Brugada syndrome patients. Arrhythmic events were classified as “early” if they occurred within 26 months of implantation, and “late” if after. A data‐driven cut‐off of 26 months was identified using the maximally selected rank statistics method over the total cohort, as explained in Section 2.6.

Appropriate diagnoses were categorized as AF, SVT, pauses, and VT.

“Atrial fibrillation” included both episodes of diagnosed AF and atrial flutter (AFLU), regardless of the duration, according to AF guidelines [17].

“Supraventricular tachycardias” included all SVT besides AF and AFLU, lasting for more than 30 s.

“Pauses” included both atrio‐ventricular conduction diseases (from BAV II grade Type 2 to BAV III grade) and sick sinus syndrome (RR interval > 3 s).

“Ventricular tachycardias” included both nonsustained (run of consecutive ventricular beats > 100 bpm persisting for 3 beats to 30 s) and sustained (continuous VT for at least 30 s, or which requires an intervention for termination) ventricular arrhythmias [16].

### 2.3 Device Implantation and Remote Control

According to the centers protocol, patients undergoing ILR implantation received either Reveal LINQ or LINQ II (Medtronic Inc., Minneapolis, MN, USA), Confirm RX or ASSERT IQ (Abbott Laboratories, Chicago, IL, USA), LUX‐Dx I or LUX‐Dx II (Boston Scientific, USA), and Biomonitor IIIm or Biomonitor IV (Biotronik SE & Co. KG, Berlin, Germany). Implant site was selected after pre‐implant electro‐anatomical surface mapping of the precordial zone to improve R‐wave sensing amplitude [18]. Device programming followed manufacturer default settings. All patients received RM with automatic arrhythmia detection. The automatic alerts by the ILR and patient‐triggered recordings due to symptoms were evaluated by two independent clinicians (L. C. and B. B.) and adjudicated by a third clinician (A. R.) in case of disagreement.

### 2.4 Endpoints

The primary endpoint was the “time to diagnosis” for each implant indication and the incidence of the specific diagnosis of interest for each clinical indication. Occurrence of diagnoses of interest was compared between “early” and “late” follow‐up periods: the number needed to follow to obtain a diagnosis (NNF) was calculated. Moreover, optimal follow‐up time was calculated for each indication, as described in Section 2.6.

The secondary endpoint was to evaluate “time to diagnosis” for any of the four target diagnoses across all four implant indications.

### 2.5 Clinical Impact Assessment

In addition to diagnostic yield, we evaluated the clinical impact of ILR‐detected diagnoses. Clinical impact was defined as any change in patient management directly triggered by the diagnosis of interest, including:

- Initiation of oral anticoagulation (for AF detection in stroke patients).
- Pacemaker implantation (for pauses in patients with syncope).
- Implantable cardioverter‐defibrillator (ICD) implantation and type of ICD chosen (for VT detection in Brugada patients).
- Antiarrhythmic drug initiation or catheter ablation (for SVT or AF in patients with palpitations).

Clinical impact was assessed through retrospective review of medical records and device reports. The proportion of diagnoses leading to actionable therapeutic interventions was calculated for each indication and compared between early (≤ 26 months) and late (> 26 months) follow‐up periods.

This analysis aimed to determine whether prolonged monitoring not only increased diagnostic yield but also translated into meaningful changes in patient management.

### 2.6 Statistical Analysis

All variables were first tested for normality with the Shapiro–Wilk test. Normally distributed variables were described as mean ± standard deviation and the groups were compared through paired or unpaired *t*‐test as appropriate. Non‐normally distributed variables were described as median [IQR] and compared by the Mann–Whitney test or the Wilcoxon signed‐rank test as appropriate. The categorical variables were described as frequencies (percentages) and compared by *χ* ^2^ test or Fisher's exact test as appropriate.

Time‐to‐event analysis was performed using Kaplan–Meier survival curves. Differences between groups were evaluated using the log‐rank test. Separate curves were generated for overall incidence and indication‐specific “diagnosis of interest.”

To determine the optimal follow‐up threshold that discriminated high versus low event rates, maximally selected rank statistics (maxstat) were applied. This data‐driven approach identified a cut‐off point that maximized the separation of survival functions and minimized *p* values, with correction for multiple testing. To ensure comparability across clinical indications, early versus late analyses were uniformly performed using the global maxstat cutoff derived from the overall cohort, whereas indication‐specific cutoffs were calculated to identify the optimal monitoring duration for each individual indication. Both analyses were performed taking in consideration only the diagnoses of interest.

Because maximally selected rank statistics identify the time point that best separates patients with and without subsequent diagnoses but may be less intuitive for individual patient counseling, we additionally calculated descriptive diagnostic accumulation thresholds. Specifically, for each implant indication, we determined the time point by which 90% and 95% of the observed diagnoses of interest had occurred. These thresholds were calculated using only patients with a diagnosis of interest and valid time‐to‐diagnosis data, according to the predefined indication‐specific diagnoses of interest. For patients implanted for palpitations or Brugada syndrome, overlapping diagnoses of interest were counted only once, using the earliest qualifying diagnosis. These descriptive thresholds were reported alongside the maxstat‐derived cutoffs and should be interpreted as the time by which 90% or 95% of observed diagnoses had occurred, rather than as absolute exclusion thresholds for future diagnoses.

To compare the incidence of arrhythmic events of interest for single indication before and after optimal follow‐up threshold we calculated event rates per 10 patient‐month and NNF, performed *χ* ^2^ tests on 2 × 2 contingency tables, assessing event occurrence differences in “early” and “late” follow‐up time. A *p* value less than 0.05 was considered statistically significant. The significance level was set at 5% and all tests were two‐tailed.

NNF was calculated as the reciprocal of the incidence rate (events per patient‐month), representing the number of patients who need to be followed for 1 month to detect one event.

Data were analyzed through Statistical Package for Social Sciences v.22 software (SPSS Inc., Chicago, Illinois, USA) and R (version 4.5.0) with the “survminer” package for survival modeling.

## 3 Results

### 3.1 Study Population

A total of 1734 consecutive patients undergoing ILR implantation at 5 Italian centers were included in the analysis. Baseline characteristics are summarized in Table 1. The median age was 64 years [range 8–86], and 1127 patients (65.0%) were male. The indication for ILR implantation was unexplained syncope in 856 patients (49.4%), cryptogenic stroke in 496 (28.6%), palpitations in 269 (15.5%), and Brugada syndrome in 113 (6.5%). Most patients received a Medtronic Reveal LINQ or LINQ II device (*n* = 1263; 72.8%), followed by Biotronik BioMonitor IIIm or BioMonitor IV (*n* = 317; 18.3%), Boston Scientific LUX‐Dx I or LUX‐Dx II (*n* = 103; 5.9%), and Abbott Confirm RX or Assert‐IQ (*n* = 51; 2.9%). Median follow‐up was 34 months [range 1–80], and the median time to any appropriate arrhythmic diagnosis was 7 months [range 1–75].

**Table 1:** Baseline characteristics.

General characteristics	Total
Total number	1734
Age, years, median [range]	64 (8–86)
Male gender, number (%)	1127 (65)
Female gender, number (%)	607 (35)
*Device manufacturer, number (%)*	
Medtronic (Reveal linq + Linq II)	1263 (72.8)
Biotronik (Biomonitor IIIm + Biomonitor IV)	317 (18.3)
Boston Scientific (Lux‐Dx I + Lux‐Dx II)	103 (5.9)
Abbott (Confirm‐RX + Assert‐IQ)	51 (2.9)
*ILR's indication, number (%)*	
Syncope	856 (49.4)
Palpitations	269 (15.5)
Cryptogenic stroke	496 (28.6)
Brugada	113 (6.5)
Follow‐up, months, median [range]	34 (1–80)
Time to diagnosis, median [range]	7 (1–75)
*ILR's appropriate diagnoses, number (%)*	
Pauses	339 (19.5)
Ventricular tachycardias	28 (1.6)
Supraventricular tachycardias	100 (5.8)
Atrial fibrillation	193 (11.1)
Sensing, mean [SD], mV	0.6 ± 0.3

During follow‐up, 339 patients (19.5%) had pauses, 193 (11.1%) had AF, 100 (5.8%) had SVT, and 28 (1.6%) had VT. The distribution of arrhythmic diagnoses differed according to the original indication for ILR implantation, supporting the use of indication‐specific diagnoses of interest in the subsequent analyses (Figure 1).

**Figure 1:**
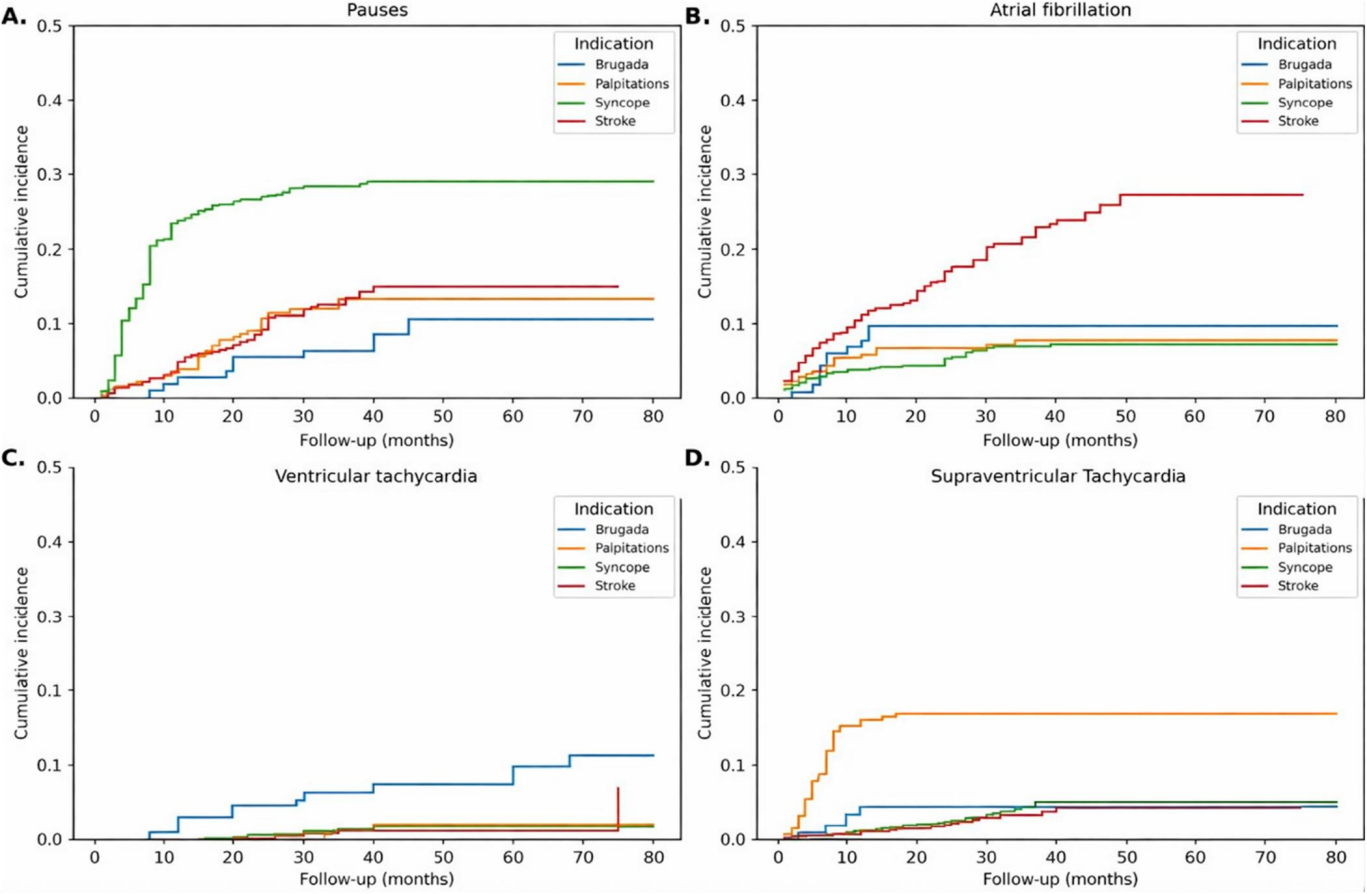
Cumulative incidence of specific arrhythmic diagnoses stratified by ILR indication. Kaplan–Meier curves showing time to first diagnosis of (A) pauses, (B) atrial fibrillation (AF), (C) ventricular tachycardia (VT), and (D) supraventricular tachycardia (SVT), stratified by implant indication (Brugada syndrome, palpitations, syncope, and stroke). Curves represent cumulative incidence over follow‐up, with censoring at last available follow‐up for patients without the specific arrhythmic event. Each panel illustrates the probability of detecting the corresponding arrhythmia over time within each indication group.

### 3.2 Overall Diagnostic Yield and Time to Diagnosis

The cumulative incidence of ILR‐detected arrhythmic diagnoses differed substantially according to implant indication (Figure 2). Across the overall cohort, maximally selected rank statistics identified 26 months as the optimal global threshold separating patients with and without a diagnosis of interest. Accordingly, diagnoses occurring at or before 26 months were classified as early, whereas diagnoses occurring after 26 months were classified as late (Figure 3).

**Figure 2:**
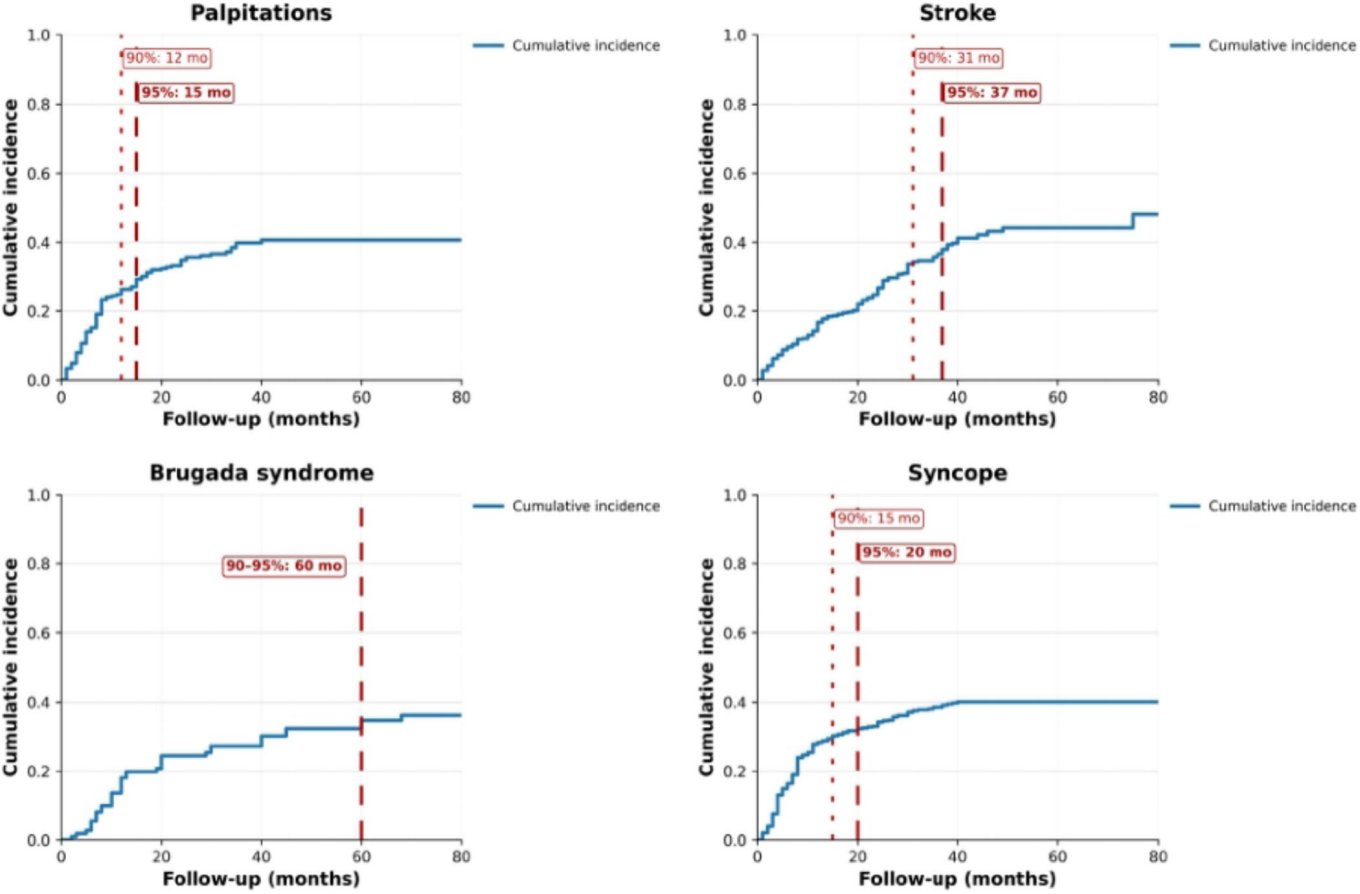
Cumulative incidence of events by ILR Indication. Kaplan–Meier curves showing the cumulative incidence of diagnoses detected by implantable loop recorders (ILRs) over time, stratified by indication: syncope, palpitations, cryptogenic stroke, and Brugada syndrome. The curves illustrate that most events occur within the first 26 months post‐implantation, except for Brugada syndrome, where diagnostic events are distributed more evenly over time. Red vertical lines indicate the time points at which 90% and 95% of observed diagnoses of interest had occurred for each indication. For Brugada syndrome, the 90% and 95% thresholds coincided at 60 months.

**Figure 3:**
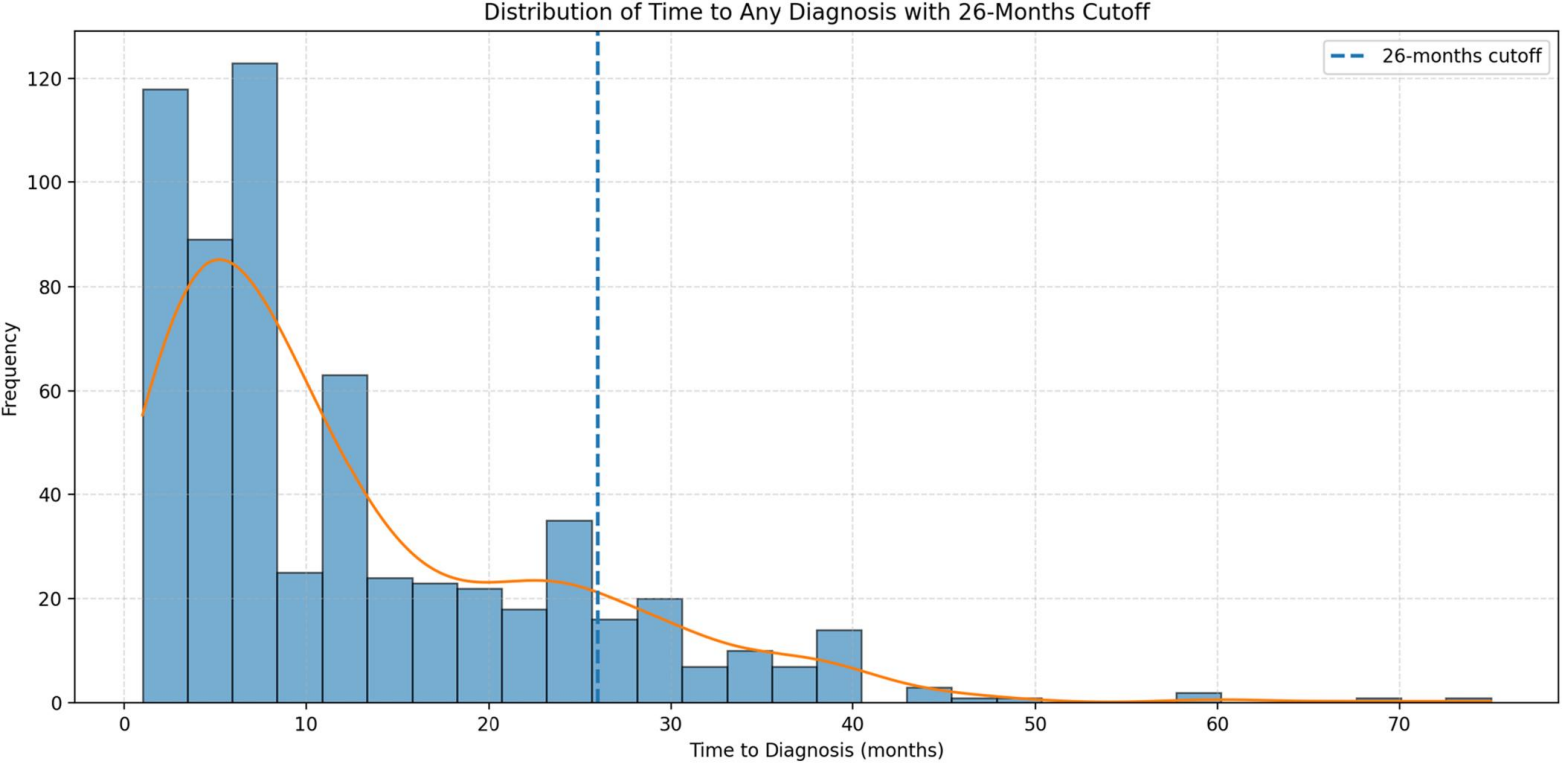
Distribution of time to first arrhythmic event in patients monitored with implantable loop recorders. The histogram displays the number of patients (frequency) who experienced their first arrhythmic event within each time interval, while the overlaid kernel density estimate (KDE) curve illustrates the smoothed probability distribution of event timing. A clear clustering of events is observed within the early follow‐up period. The red dashed line indicates the 26‐month cutoff used in the statistical analysis to compare incidence before and after this time point.

When indication‐specific diagnoses of interest were considered, the optimal monitoring window varied markedly across clinical groups (Table 2). The optimal cutoff was 14 months for pauses in patients implanted for syncope, 13 months for SVT or AF in patients implanted for palpitations, and 16 months for AF in patients implanted after cryptogenic stroke. In contrast, patients with Brugada syndrome showed a substantially longer optimal diagnostic window, with the greatest separation in diagnostic yield observed at 55 months for VT or pauses. These findings indicate that the temporal pattern of ILR diagnostic yield is not uniform across indications, but instead follows distinct indication‐specific trajectories.

**Table 2:** Optimal follow‐up cutoffs by clinical indication based on diagnoses of interest.

Indication	Diagnosis of interest	Optimal cutoffs, maxstat (months)	Time by which 90% occurred	Time by which 95% occurred	*p*
Syncope	Pauses	14	15	20	< 0.001
Stroke	AF	16	31	37	< 0.001
Palpitations	SVT and AF	13	12	15	< 0.001
Brugada	VT and Pauses	55	60	60	< 0.001

*Note:* This table reports, for each clinical indication, the maxstat‐derived optimal follow‐up cutoff and the descriptive time points by which 90% and 95% of the observed diagnoses of interest had occurred. Diagnoses of interest were defined a priori as follows: pauses for syncope, atrial fibrillation (AF) for cryptogenic stroke, supraventricular tachycardia (SVT) or AF for palpitations, and ventricular tachycardia (VT) or pauses for Brugada syndrome. Maxstat cutoffs were determined using maximally selected rank statistics and identify the time point that best separated patients with and without subsequent diagnoses. The 90% and 95% thresholds were calculated among patients with a diagnosis of interest and valid time‐to‐diagnosis data, with overlapping diagnoses counted only once using the earliest qualifying event. These descriptive thresholds indicate when 90% or 95% of observed diagnoses had already occurred and should not be interpreted as absolute exclusion of future diagnoses. The *p* value indicates the statistical significance in event occurrence before and after the maxstat‐derived cutpoint.

To provide a more clinically intuitive estimate of diagnostic accumulation over time, we also calculated the time points by which 90% and 95% of observed diagnoses of interest had occurred for each indication (Table 2). In patients implanted for syncope, 90% and 95% of pause diagnoses had occurred by 15 and 20 months, respectively. In patients implanted for palpitations, 90% and 95% of SVT or AF diagnoses had occurred by 12 and 15 months, respectively. In patients implanted after cryptogenic stroke, AF diagnoses accumulated over a longer period, with 90% and 95% of observed AF diagnoses occurring by 31 and 37 months, respectively. In patients with Brugada syndrome, diagnostic accumulation was most delayed, with both the 90% and 95% thresholds reached at 60 months. These descriptive thresholds complement the maxstat‐derived cutoffs by providing clinically interpretable time points for patient counseling, while not implying absolute exclusion of future diagnoses beyond these time points.

The comparison of early and late diagnostic efficiency further confirmed these differences (Table 3). In syncope and palpitations, the yield of diagnoses of interest declined sharply after 26 months. In cryptogenic stroke, AF detection also decreased after 26 months, although late diagnoses continued to occur. By contrast, in Brugada syndrome, the number of diagnoses of interest was identical before and after 26 months, with only a modest change in event rate and number needed to follow.

**Table 3:** Diagnosis of interest analysis of ILR efficiency before and after 26 months.

Indication	Events ≤ 26 m	Events > 26 m	Rate ≤ 26 m (per 10 pt‐month)	Rate > 26 m (per 10 pt‐month)	NNF ≤ 26 M (95% CI)	NNF > 26 M (95% CI)	*p*
Brugada *n* = 113	11	11	0.038	0.027	26.04 (14.55–52.16)	31.80 (20.57–65.72)	0.929
Stroke *n* = 496	85	20	0.070	0.039	14.21 (11.50–17.80)	25.92 (16.78–42.43)	< 0.001
Palpitations *n* = 269	63	2	0.093	0.006	10.78 (8.42–14.03)	177.75 (49.21–1467.74)	< 0.001
Syncope *n* = 856	226	6	0.113	0.007	8.82 (7.74–10.09)	147.92 (67.96–403.06)	< 0.001

*Note:* This table summarizes the number and rate of first arrhythmic events detected by implantable loop recorders (ILRs) in a 1734‐patient cohort, stratified by clinical indication and relevant diagnosis type. For each indication, only clinically appropriate arrhythmias were considered: ventricular tachycardia (VT) and pauses for Brugada syndrome; atrial fibrillation (AF) for cryptogenic stroke; AF and supraventricular tachycardia (SVT) for palpitations; and pauses for syncope. The table compares event incidence and diagnostic efficiency during early (≤ 26 months) versus late (> 26 months) follow‐up periods, expressed as event rates per 10 patient‐month, number needed to follow (NNF), and associated *p* values from *χ* ^2^ testing. These results highlight indication‐specific patterns of ILR yield over time, emphasizing the value of personalized follow‐up strategies.

### 3.3 Indication‐Specific Diagnostic Yield

#### 3.3.1 Syncope

Among 856 patients implanted for unexplained syncope, the median follow‐up was 30 months [range 16–80]. Overall, 310 patients (36.2%) experienced an arrhythmic diagnosis during follow‐up. Pauses represented the most frequent arrhythmic finding and the predefined diagnosis of interest for this indication, occurring in 232 patients (27.2%). AF was detected in 53 patients (6.2%), SVT in 34 (4.0%), and VT in 9 (1.1%) (Figure 4).

**Figure 4:**
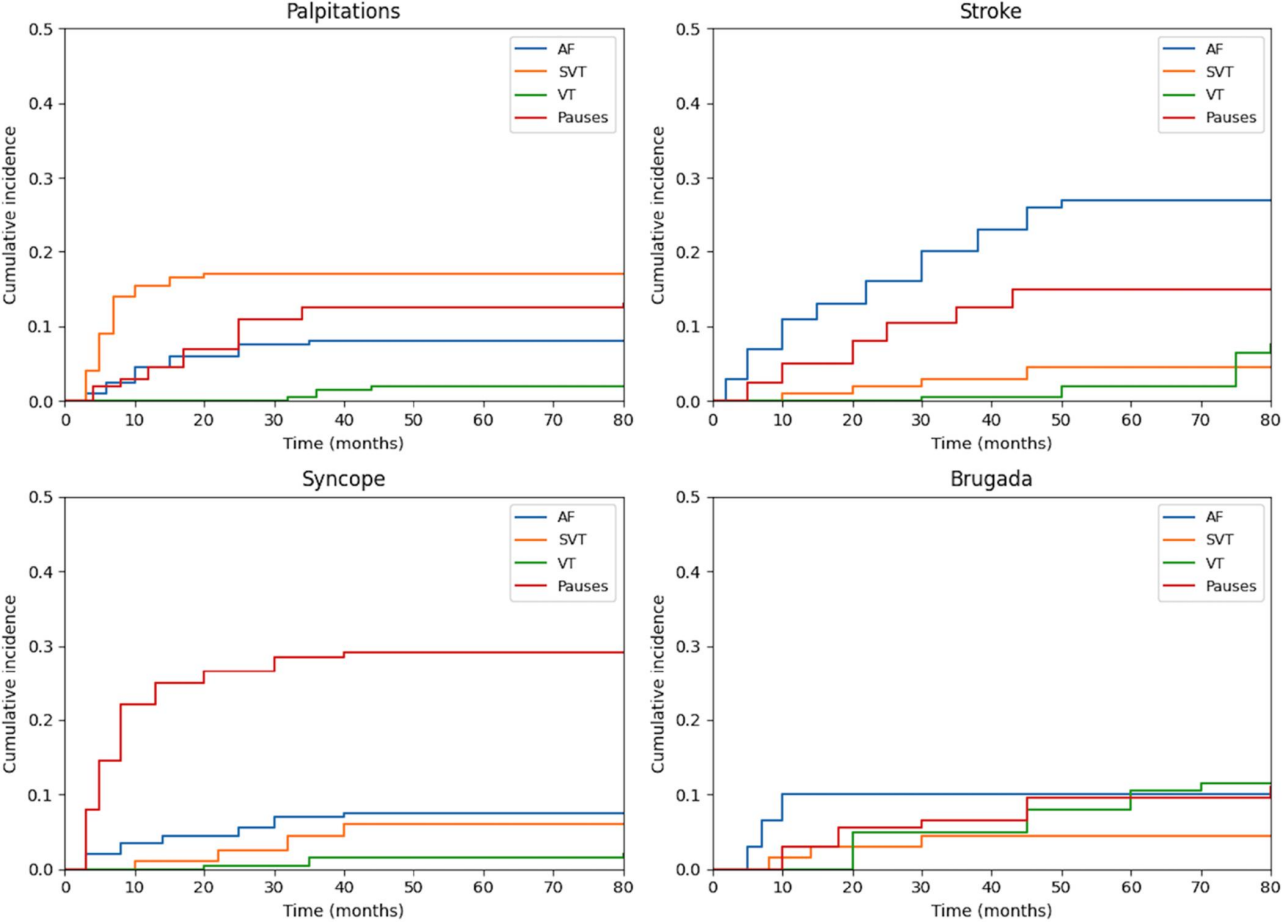
Time to diagnosis curves for specific arrhythmias across ILR indications. Kaplan–Meier curves representing time to first detection of each arrhythmia type (pauses, atrial fibrillation (AF), supraventricular tachycardia (SVT), and ventricular tachycardia (VT)) across the study population. Each panel corresponds to a different arrhythmia subtype, showing cumulative incidence from ILR implantation to event detection. The analysis highlights that pauses and AF are the most frequent diagnoses, occurring predominantly in the early follow‐up period, while VT events occur less frequently but may present later, particularly in Brugada syndrome patients.

The optimal indication‐specific cutoff for detection of pauses in patients with syncope was 14 months (Table 2). Using the global 26‐month threshold, 226 pause diagnoses occurred during early follow‐up, whereas only 6 occurred after 26 months. The event rate decreased from 0.113 to 0.007 events per 10 patient‐months, and the number needed to follow increased from 8.82 to 147.92. This reduction in diagnostic efficiency was statistically significant (*p* < 0.001; Table 3). Among the nine patients implanted for syncope in whom VT was detected, five developed nonsustained and four sustained VT. None of them developed VF. Only two patients were found to have structural heart disease. VT detection led to management change in only four patients, including ICD implantation and, in one case, VT ablation. Importantly, these cases were clinically distinct from the broader unexplained syncope population, and patients with persistent suspicion of tachyarrhythmic syncope should be considered individually.

#### 3.3.2 Palpitations

Among 269 patients implanted for palpitations, the median follow‐up was 35 months [range 3–80]. Overall, 106 patients (39.4%) experienced an arrhythmic diagnosis. SVT was detected in 45 patients (16.7%), AF in 24 (8.9%), pauses in 34 (12.6%), and VT in 3 (1.1%) (Figure 4). For this indication, SVT and AF were considered diagnoses of interest.

The optimal indication‐specific cutoff for diagnosis of SVT or AF was 13 months (Table 2). After accounting for patients with overlapping SVT and AF episodes, 65 patients (24.1%) had a diagnosis of interest. Of these, 63 diagnoses occurred within the first 26 months and only 2 occurred thereafter. The event rate declined from 0.093 to 0.006 events per 10 patient‐months, while the number needed to follow increased from 10.78 to 177.75 after 26 months (*p* < 0.001; Table 3).

#### 3.3.3 Cryptogenic Stroke

Among 496 patients implanted after cryptogenic stroke, the median follow‐up was 34 months [range 20–75]. Overall, 178 patients (35.9%) experienced an arrhythmic diagnosis. AF, the predefined diagnosis of interest for this indication, was detected in 105 patients (21.2%). Pauses were detected in 61 patients (12.3%), SVT in 16 (3.2%), and VT in 5 (1.0%) (Figure 4).

The optimal indication‐specific cutoff for AF detection after cryptogenic stroke was 16 months (Table 2). Using the global 26‐month threshold, 85 AF diagnoses occurred during early follow‐up and 20 occurred during late follow‐up. The event rate decreased from 0.070 to 0.039 events per 10 patient‐months, and the number needed to follow increased from 14.21 to 25.92 after 26 months. This difference was statistically significant (*p* < 0.001; Table 3), indicating a decline in diagnostic efficiency over time despite persistent late AF detection.

#### 3.3.4 Brugada Syndrome

Among 113 patients implanted for Brugada syndrome, the median follow‐up was 70 months [range 26–80]. Overall, 38 patients (33.6%) experienced an arrhythmic diagnosis during follow‐up. Pauses occurred in 11 patients (9.7%), VT in 11 (9.7%), AF in 11 (9.7%), and SVT in 5 (4.4%) (Figure 4). For this indication, VT and pauses were considered diagnoses of interest.

The optimal indication‐specific cutoff for VT or pause detection was 55 months (Table 2). Overall, 22 diagnoses of interest were observed in 16 patients (14.2%), including VT in 11 patients and pauses in 11 patients; 6 patients experienced both VT and pauses. In contrast to the other indications, diagnoses were evenly distributed over time, with 11 diagnoses occurring within the first 26 months and 11 occurring after 26 months. The event rate decreased only modestly from 0.038 to 0.027 events per 10 patient‐months, and the number needed to follow changed from 26.04 to 31.80. The difference between early and late follow‐up was not statistically significant (*p* = 0.929; Table 3), indicating sustained diagnostic yield during prolonged monitoring in this subgroup.

### 3.4 Clinical Impact of ILR‐Detected Diagnoses

The therapeutic consequences of ILR‐detected diagnoses differed according to the original indication for implantation and the type of arrhythmia detected (Figure 5). In patients implanted for syncope, all patients with ILR‐detected pauses underwent permanent pacemaker implantation. Among the six patients with late pause detection after 26 months, pauses were recorded after recurrent syncopal episodes in which no concomitant arrhythmia had previously been documented. Three of these six patients experienced recurrent syncope even after pacemaker implantation. In patients implanted for palpitations, all patients with ILR‐detected SVT underwent catheter ablation. Among patients with AF, 15 patients (60%) underwent pulmonary vein isolation, whereas 9 patients (40%) were treated with antiarrhythmic drug therapy. Late arrhythmic diagnoses in this group were not associated with concomitant symptoms at the time of detection.

**Figure 5:**
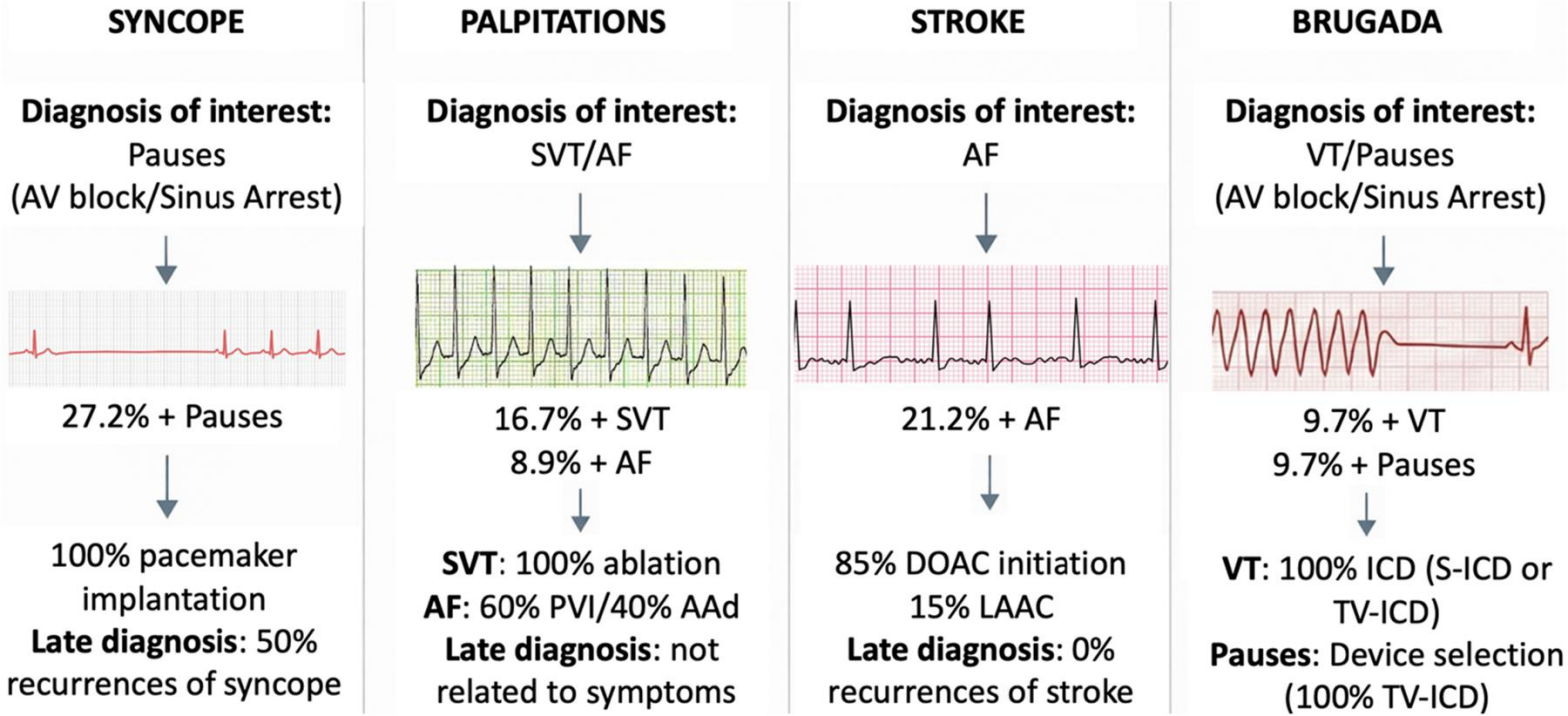
Indication‐specific therapeutic consequences of ILR‐detected arrhythmias. This schematic summarizes the clinical implications of arrhythmias detected by implantable loop recorders (ILRs) according to the primary indication for implantation. In patients with syncope, detection of significant pauses (including atrioventricular block or sinus arrest) resulted in permanent pacemaker implantation in all cases. In patients with palpitations, supraventricular tachycardia (SVT) led to catheter ablation, whereas atrial fibrillation (AF) prompted rhythm‐control strategies, including pulmonary vein isolation (PVI) or antiarrhythmic drug therapy. In patients with cryptogenic stroke, ILR‐detected AF resulted in initiation of oral anticoagulation in the majority of cases or left atrial appendage closure in patients with high bleeding risk. In patients with Brugada syndrome, ventricular tachycardia (VT) led to implantable cardioverter‐defibrillator (ICD) implantation. Detection of significant pauses influenced device strategy by identifying patients requiring pacing capability, favoring transvenous ICD systems when appropriate. AAD = antiarrhythmic drugs; ICD = implantable cardioverter‐defibrillator; LAAC = left atrial appendage closure; PVI = pulmonary vein isolation; S‐ICD = subcutaneous implantable cardioverter‐defibrillator; TV‐ICD = transvenous implantable cardioverter‐defibrillator.

In patients implanted after cryptogenic stroke, ILR‐detected AF led to a change in antithrombotic management in all cases. Oral anticoagulation was initiated in 89 patients (85%), whereas 16 patients (15%) underwent left atrial appendage closure because of high bleeding risk. No recurrent ischemic stroke was observed during follow‐up among patients with ILR‐detected AF.

In patients with Brugada syndrome, all patients with ILR‐detected VT underwent ICD implantation. Detection of pauses influenced device selection by identifying patients requiring pacing capability. Six patients had both VT and pauses and received a transvenous ICD. Among the remaining five patients with pauses without documented VT, four underwent ICD implantation and one was managed conservatively because the episode was asymptomatic.

### 3.5 ILR Replacement After Battery Depletion

Overall, 121 patients (6.9%) underwent ILR replacement after battery depletion. Patients with Brugada syndrome accounted for the largest proportion of replacements (49 of 121; 40.5%), followed by syncope (*n* = 43), palpitations (*n* = 15), and cryptogenic stroke (*n* = 14).

A diagnosis of interest after ILR replacement was observed in only 8 patients (6.6%). Most post‐replacement diagnoses occurred in patients with Brugada syndrome (*n* = 5), whereas fewer were observed in patients implanted after cryptogenic stroke (*n* = 2) or for palpitations (*n* = 1). No diagnosis of interest occurred after ILR replacement in patients implanted for syncope. These findings further support an indication‐dependent approach to ILR replacement, with the lowest post‐replacement yield observed in syncope and the highest relative yield observed in Brugada syndrome.

## 4 Discussion

In this multicenter cohort of 1734 patients undergoing ILR implantation for syncope, palpitations, cryptogenic stroke, or Brugada syndrome, the diagnostic and therapeutic value of monitoring differed substantially according to the indication for implantation. The main findings are as follows. First, ILR diagnostic yield was strongly time‐dependent, with most actionable diagnoses in syncope, palpitations, and cryptogenic stroke occurring during the first 14–16 months of follow‐up. Second, late arrhythmic diagnoses were not clinically equivalent across indications: in syncope and palpitations, late findings were uncommon and often less clearly related to the index presentation, whereas in cryptogenic stroke, late AF detection could still modify antithrombotic management. Third, Brugada syndrome represented a distinct subgroup in which diagnostic yield remained sustained over prolonged follow‐up, with VT or pauses detected both early and late and with direct implications for ICD implantation or device selection. These observations support an indication‐driven approach to ILR follow‐up duration and replacement rather than a uniform strategy based primarily on device longevity.

### 4.1 Principal Findings

ILRs were originally established as a diagnostic tool for unexplained recurrent syncope, where symptom–rhythm correlation is essential to guide therapy [1, 2, 3, 4, 5, 6]. Over time, their use has expanded to broader clinical scenarios, including AF detection after cryptogenic stroke, evaluation of palpitations, and risk assessment in selected patients with Brugada syndrome [7, 8, 9, 10, 11, 12]. However, these indications differ substantially in their diagnostic target, clinical urgency, and interpretation of late arrhythmia detection. Therefore, the same monitoring duration may not provide the same clinical value across all patient groups.

Our results show that the temporal distribution of ILR‐detected diagnoses is not uniform. In patients implanted for syncope or palpitations, the diagnostic yield was concentrated early after implantation and declined sharply after the global 26‐month threshold. In contrast, patients with Brugada syndrome had a much longer optimal diagnostic window, with an indication‐specific cutoff of 55 months. Cryptogenic stroke showed an intermediate pattern: AF detection was more frequent early, but late AF remained potentially actionable. These findings extend previous reports on ILR diagnostic yield by emphasizing not only whether arrhythmias are detected, but also when they are detected and whether they remain clinically meaningful [8, 13].

This distinction is clinically relevant because increasing device longevity does not necessarily imply persistent diagnostic usefulness. Current‐generation ILRs may monitor patients for several years, but the incremental value of continued surveillance depends on the probability that a late diagnosis will change management. In this regard, our data suggest that ILR follow‐up should be tailored to the original clinical question rather than automatically prolonged until battery depletion or routinely repeated after device exhaustion.

### 4.2 Syncope and Palpitations: Time‐Limited Symptom‐Driven Monitoring

Syncope and palpitations are symptom‐driven indications in which the principal value of ILR monitoring lies in establishing a convincing relationship between symptoms and rhythm disturbance. In our cohort, patients implanted for syncope showed a clear early concentration of pause detection. The optimal cutoff for the diagnosis of pauses was 14 months; 90% and 95% of observed pause diagnoses had occurred by 15 and 20 months, respectively, and only 6 pause diagnoses occurred after 26 months, compared with 226 during the early period. This pattern is consistent with prior studies showing that ILRs are particularly useful in the early diagnostic phase of unexplained syncope, when recurrent events are most likely to reveal a bradyarrhythmic mechanism [5, 6, 19, 20].

The clinical interpretation of late pause detection in syncope requires caution. Although all patients with ILR‐detected pauses underwent pacemaker implantation, late pauses were documented after previous recurrent syncopal episodes in which no concomitant arrhythmia had been captured. Moreover, three of the six patients with late pause detection experienced recurrent syncope despite pacemaker implantation. This finding suggests that, in some patients, late bradyarrhythmias may represent associated or incidental findings rather than the dominant mechanism of the original syncopal syndrome. Therefore, when ILR monitoring remains unrevealing after an adequate early observation period, clinicians should reconsider alternative mechanisms, including reflex, orthostatic, neurologic, or noncardiac causes, rather than relying indefinitely on prolonged rhythm surveillance.

A similar concept applies to patients implanted for palpitations. In this group, the optimal diagnostic cutoff for SVT or AF was 13 months, 90% and 95% of observed SVT or AF diagnoses had occurred by 12 and 15 months, respectively, and only 2 diagnoses of interest occurred after 26 months. The marked increase in the number needed to follow after 26 months indicates a substantial reduction in diagnostic efficiency. Importantly, late arrhythmic diagnoses were not associated with concomitant symptoms at the time of detection, weakening their causal relationship with the original complaint. In patients with palpitations, a late asymptomatic episode of SVT or AF may still be clinically relevant in selected cases, but it may not explain the symptom that prompted ILR implantation.

Taken together, these findings support a time‐limited strategy for symptom‐driven ILR indications. In syncope and palpitations, prolonged monitoring after an unrevealing early phase should not be considered automatically beneficial. Rather, continuation or reimplantation should depend on recurrent symptoms, residual suspicion of arrhythmic causality, patient risk profile, and whether a new diagnosis would plausibly alter treatment.

### 4.3 Cryptogenic Stroke: Persistent Clinical Relevance but Declining Efficiency

Patients implanted after cryptogenic stroke showed a different pattern. AF detection was most frequent during the early phase, with an indication‐specific optimal cutoff of 16 months, and diagnostic efficiency declined after 26 months. Nevertheless, the descriptive accumulation thresholds showed that 90% and 95% of observed AF diagnoses occurred by 31 and 37 months, respectively, supporting the view that AF detection after cryptogenic stroke may extend beyond the statistically optimal early window. Unlike syncope and palpitations, the clinical relevance of a late diagnosis cannot be dismissed solely because it occurs later. AF detected after cryptogenic stroke may still justify a major change in secondary prevention strategy, particularly initiation of oral anticoagulation or, in selected patients with high bleeding risk, left atrial appendage closure.

This creates a “gray‐zone” indication for prolonged ILR monitoring. On one hand, our data show that the number needed to follow increased after 26 months, indicating reduced efficiency over time. On the other hand, 20 AF diagnoses occurred after 26 months, and all AF diagnoses in the stroke group led to a change in antithrombotic management. These findings are consistent with the broader rationale for extended rhythm monitoring after cryptogenic stroke or embolic stroke of undetermined source, where occult AF may be intermittent and delayed in its detection [9, 12, 21].

Therefore, in cryptogenic stroke, prolonged monitoring should probably be individualized rather than routinely discontinued or automatically extended. Factors that may support longer monitoring include older age, left atrial enlargement, frequent atrial ectopy, absence of an alternative stroke mechanism, recurrent embolic events, and a clinical profile suggesting atrial cardiopathy. Conversely, when competing noncardioembolic mechanisms are identified or bleeding risk limits therapeutic consequences, the incremental value of very prolonged surveillance may be lower. Our findings support a balanced approach: cryptogenic stroke is not equivalent to Brugada syndrome, where long‐term monitoring retained sustained yield, but it is also not equivalent to palpitations or syncope, where late diagnoses were rare or less clearly linked to the index presentation.

### 4.4 Brugada Syndrome: Sustained Value of Long‐Term Monitoring

Brugada syndrome emerged as the clearest example of an indication in which prolonged ILR monitoring may remain clinically valuable. Although the Brugada subgroup was smaller than the other groups, the temporal pattern was markedly different. A total of 22 Brugada‐related diagnoses of interest were observed in 16 patients, with exactly half of the diagnoses occurring within 26 months and half thereafter. The event rate decreased only modestly during late follow‐up, and the difference between early and late diagnostic yield was not statistically significant. The indication‐specific optimal cutoff was 55 months, substantially longer than for syncope, palpitations, or cryptogenic stroke. Consistently, both the 90% and 95% diagnostic accumulation thresholds were reached only at 60 months, further illustrating the delayed and sustained nature of clinically relevant arrhythmic detection in this subgroup.

These findings are consistent with the concept that Brugada syndrome is characterized by unpredictable arrhythmic events and that short‐term monitoring may be insufficient in selected patients [7, 16]. In this population, the clinical relevance of late arrhythmia detection is high. Detection of VT may support ICD implantation, while documentation of clinically significant pauses may influence the selection between a subcutaneous and transvenous ICD system by identifying patients who may require pacing capability. This distinction is important because device choice in Brugada syndrome must balance the risk of ventricular arrhythmias, bradyarrhythmias, long‐term lead complications, and the patient's age and expected device exposure.

The BruLoop study highlighted the potential role of ILRs in the management of selected Brugada syndrome patients [7]. Our data add a temporal dimension to this concept by suggesting that the diagnostic value of ILR monitoring in Brugada syndrome may persist well beyond the period in which other indications show sharply reduced yield. In selected Brugada patients, particularly those with unexplained syncope, negative or inconclusive risk stratification, or uncertainty regarding ICD indication or device type, extended surveillance may therefore be clinically justified (Central Illustration 1).

**Central Illustration 1:**
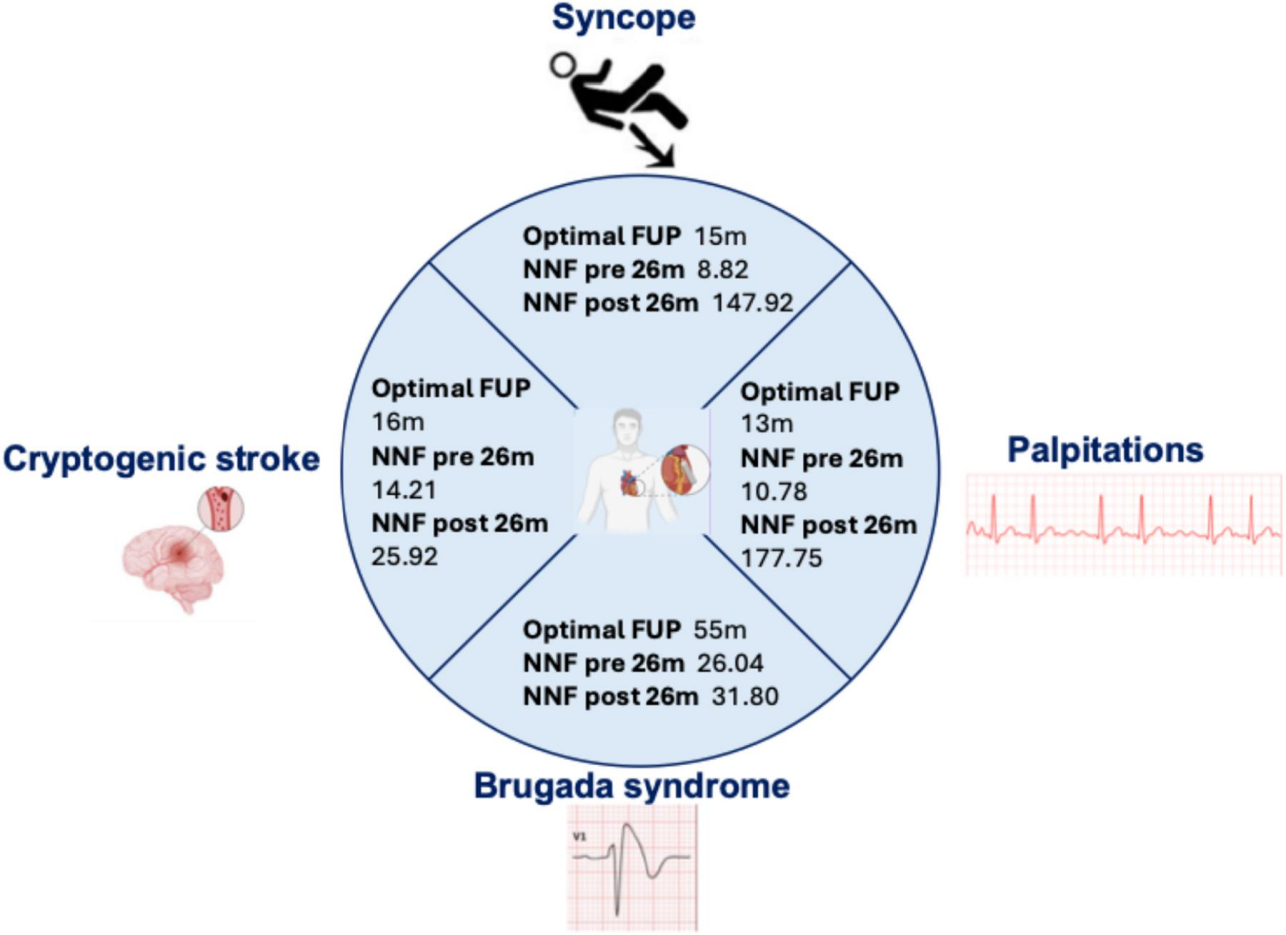
Indication‐specific value of prolonged implantable loop recorder monitoring. The diagnostic and clinical value of implantable loop recorder monitoring differs according to implant indication. In syncope and palpitations, actionable diagnoses are concentrated early after implantation, and prolonged monitoring after an unrevealing early phase has limited incremental value. In cryptogenic stroke, atrial fibrillation detection is most frequent early but may remain clinically actionable later, supporting individualized monitoring duration. In Brugada syndrome, ventricular arrhythmias and pauses may occur late and retain major therapeutic implications, supporting extended surveillance in selected patients.

### 4.5 Implication for Reimplantation and Resource Utilization

One of the practical implications of this study concerns ILR replacement after battery depletion. In the overall cohort, only 121 patients underwent ILR replacement, and a diagnosis of interest after replacement was observed in 8 patients. Importantly, this post‐replacement yield was not evenly distributed across indications. Most post‐replacement diagnoses occurred in Brugada syndrome, whereas no diagnosis of interest was observed after replacement in patients implanted for syncope.

These findings argue against routine ILR reimplantation after battery depletion in all patients. In syncope and palpitations, especially when early monitoring is unrevealing and symptoms have not recurred with convincing arrhythmic suspicion, reimplantation is likely to provide limited incremental value. In cryptogenic stroke, reimplantation may be reasonable in selected patients with persistent suspicion of occult AF and a clear therapeutic consequence of detection. In Brugada syndrome, replacement may be more defensible because late VT or pauses may retain major prognostic and therapeutic implications.

From a health‐system perspective, an indication‐driven strategy may improve the efficiency of RM programs. ILRs generate a continuous flow of transmissions, alerts, adjudication workload, outpatient visits, and downstream procedures. Prior studies have emphasized the diagnostic and economic value of ILR strategies in selected populations, particularly syncope and cryptogenic stroke [2, 6, 21, 22]. However, the value of prolonged monitoring depends on maintaining a favorable relationship between diagnostic yield, clinical actionability, and resource use. Avoiding low‐yield prolonged surveillance in populations with diminishing diagnostic efficiency, while preserving extended monitoring in groups with sustained clinical value, may represent a more rational use of ILR technology.

### 4.6 Limitations

This study has several limitations. First, its retrospective and observational design precludes standardized management decisions and limits the ability to infer causality between ILR‐detected arrhythmias and clinical outcomes. Second, although the overall cohort was large, some subgroups, particularly Brugada syndrome, were relatively small, which may limit statistical power and precision for indication‐specific estimates. Third, the global 26‐month cutoff was derived using maximally selected rank statistics within the study population and requires external validation.

Fourth, the definition of “diagnosis of interest” was indication‐driven and clinically pragmatic, but it was not externally validated. This approach was chosen to reflect the arrhythmias most likely to explain or modify management for each implant indication; however, some arrhythmias outside these predefined categories may still have clinical importance in individual patients. Fifth, pre‐implant diagnostic work‐up was not fully uniform across centers, reflecting real‐world practice but introducing potential heterogeneity. Sixth, long‐term clinical outcomes after ILR‐guided management, including mortality, recurrent syncope burden, recurrent stroke, arrhythmic events after ICD implantation, and cost‐effectiveness, were not systematically assessed.

Finally, the decision to replace an ILR after battery depletion was not randomized and may have been influenced by physician preference, patient characteristics, residual clinical concern, or center‐specific practice. Therefore, the post‐replacement yield should be interpreted as hypothesis‐generating. Prospective studies are needed to validate indication‐specific monitoring durations and to determine whether tailored ILR follow‐up and replacement strategies improve clinical outcomes, resource utilization, and cost‐effectiveness compared with uniform long‐term surveillance.

## 5 Conclusion

The diagnostic and therapeutic value of ILR monitoring is strongly time‐ and indication‐dependent. In patients implanted for syncope or palpitations, actionable diagnoses are concentrated in the early follow‐up period, and prolonged monitoring after an unrevealing early phase has limited incremental value. In cryptogenic stroke, AF detection was most frequent early but could still lead to changes in antithrombotic management later, supporting an individualized “gray‐zone” approach to prolonged monitoring. In Brugada syndrome, VT and pauses may occur late and retain major prognostic and therapeutic implications, supporting extended surveillance in selected patients. These findings support an indication‐driven approach to ILR follow‐up and reimplantation decisions, aligning monitoring duration with the likelihood of clinically meaningful arrhythmia detection rather than device longevity alone.

## Funding

The authors have nothing to report.

## Ethics Statement

The study was approved by the institutional ethics committees of the participating centers and was conducted in accordance with the Declaration of Helsinki.

## Consent

All patients provided informed consent for ILR implantation and use of anonymized clinical data for research purposes.

## Conflicts of Interest

The authors declare no conflicts of interest.

## Data Availability

The data underlying this article will be shared on reasonable request to the corresponding author, subject to institutional approvals and applicable privacy regulations.
